# Feature Selection Applying Statistical and Neurofuzzy Methods to EEG-Based BCI

**DOI:** 10.1155/2015/781207

**Published:** 2015-04-21

**Authors:** Juan-Antonio Martinez-Leon, Jose-Manuel Cano-Izquierdo, Julio Ibarrola

**Affiliations:** Universidad Politécnica de Cartagena, Campus Muralla del Mar, Calle Doctor Fleming S/N, 30202 Cartagena, Spain

## Abstract

This paper presents an investigation aimed at drastically reducing the processing burden required by motor imagery brain-computer interface (BCI) systems based on electroencephalography (EEG). In this research, the focus has moved from the channel to the feature paradigm, and a 96% reduction of the number of features required in the process has been achieved maintaining and even improving the classification success rate. This way, it is possible to build cheaper, quicker, and more portable BCI systems. The data set used was provided within the framework of BCI Competition III, which allows it to compare the presented results with the classification accuracy achieved in the contest. Furthermore, a new three-step methodology has been developed which includes a feature discriminant character calculation stage; a score, order, and selection phase; and a final feature selection step. For the first stage, both statistics method and fuzzy criteria are used. The fuzzy criteria are based on the S-dFasArt classification algorithm which has shown excellent performance in previous papers undertaking the BCI multiclass motor imagery problem. The score, order, and selection stage is used to sort the features according to their discriminant nature. Finally, both order selection and Group Method Data Handling (GMDH) approaches are used to choose the most discriminant ones.

## 1. Introduction

Brain-computer interface (BCI) systems capture brain signals and decode them with the purpose of interacting with external devices without any muscular or physical intervention. Well-known examples are motor imagery tasks due to their importance in applications for severely motor impaired people. Likewise, other patterns can also be recognized within the brain signals, including word generation or object rotation. These patterns can be transformed to distinguishable signals and then to external commands or actions [1].

Technologically, most of the BCI mechanisms are based on electroencephalogram (EEG) techniques, where the sensors detecting the electric potentials originated by the neurons are placed on the scalp of the user [2]. Among the noninvasive technologies, where examples like magnetoencepahlography (MEG), position emission tomography (PET), or functional Magnetic Resonance Imaging (fMRI) systems can be considered, the main benefits of the EEG approach are the cost and the portability, making its use feasible in environments out of the laboratory. These systems show major benefits when being compared with invasive methods like electrocorticopgraphy (ECoG) [3] due to the fact that no brain surgery is being required to set up the montage.

According to how the brain signals get activated, two different paradigms can be distinguished [4, 5]. On the one hand, they can be produced spontaneously by human specific thoughts without any sensory stimulus. Examples of this comprise the detection EEG rhythms (*δ*: 0–4 Hz, *θ*: 4–8 Hz, *α*: 8–12 Hz, *μ*: 8–13, and *β*: 13–30 Hz) [6], slow cortical potentials (SCP), or event-related desynchronization (ERD)/event-related synchronization (ERS). On the other hand, the brain signals can be evoked by external stimulation, without prior training. Examples of the use of this method are the applications based on P300 [7], Steady-State Visual Evoked Potential (SSVEP), or hybrid BCI systems combining both of them [8–10].

Because the recorded brain signals are so small in amplitude, EEG devices in particular present a very low signal to noise ratio (SNR). For this reason, any interference coming from sources such as eye movement, eye-blink, muscular movements, teeth clash, or the heart rhythm deeply affects the quality of the measured signal, which can prevent the decoding system from properly recognizing the intention of the user. As a consequence, an effort to improve the spatial filtering methods [11], the feature extraction techniques [12, 13], and the classification algorithms [14–16] has been undertaken by the scientific community.

In recent years, there has been increasing interest in minimizing the number of channels and features used by the classification algorithms. Yang et al. [17] identify three major drawbacks when using data from all channels by applying conventional ANNs, which can be extended to any EEG classifier: irrelevant features adding noise to the data and an increase in the complexity of the model and more computational burden. Other limitations can be added when considering the functional side and the cost of an EEG system. Tam et al. [18] measured the time to set up a 32-channel montage, achieving a total of 10–15 minutes when being done by an experienced operator (between 20 and 30 seconds per sensor). Regarding the cost, a public pricing list is available in [19] where doubling the number of electrodes seems to increase the overall cost of the system by around 25%.

There are a large number of published studies describing different approaches to feature and channel selection. These approaches comprise both wrapper and filter methods of feature selection. The most popular methods are Genetic Algorithms (GA) [17, 20, 21], Distinction Sensitive Learning Vector Quantizer (DSLVQ) [22], Mutual Information algorithms (MI) [23], Fisher Criterion (FC) [1, 18, 24] methods, and Common Spatial Pattern (CSP) techniques [25, 26]. In addition, other approaches based on wavelet packet decomposition (WPD) [1] and combinations or evolutions of the previous methods like Rayleigh Coefficient and Genetic Algorithms [27], Sparse CSP (SCSP), Robust Sparse CSP (RSCSP) [28], or Mutual Information improvements as shown in [29, 30] have also been presented to the research community. Common to all of the studies, a direct relationship between the selected sensors and the expected cortical areas is shown, although different level of success has been attained.

In [18], a work is presented where the intention of movement detection is studied in stroke patients. The selection of a minimum number of electrodes allowing it to maintain a high success rate is suggested. For that purpose, two channel selection methods are proposed: Fisher Criterion and Support Vector Machine-Recursive Feature Elimination. From an initial number of 50 channels, it demonstrated that it is possible to select 12 electrodes while maintaining the performance. The Common Spatial Pattern algorithm has also been used to define methods of channel selection [25, 26]. In both works, data from the BCI Competition is used and it is shown that it is possible to maintain and even improve the classification performance considerably reducing the number of used channels. In both scenarios, the channel selection is done by using the data in raw format before the feature extraction stage. An even more recent approach has been developed by Aler et al. [31], who present a new method for classification and feature selection, thus improving the preprocessing stage for the same data set and problem used in this paper.

Although extensive research has been carried out on feature selection, most of the available research has focused on reducing the number of channels required instead of the number of individual features. Also, no single study exists which adequately covers the result of implementing Statistical and Fuzzy approaches.

In Cano-Izquierdo et al. [14], the dFasArt is proposed as a neurofuzzy model for the self-organised learning whose defined clusters are determined by the weights of the units, which can be interpreted as rules on fuzzy sets. The connections between the units of the model and the value of the weights define a Fuzzy Logic System (FLS). Among the characteristics of the dFasArt, it is worth highlighting the way the clustering works according to the incoming values and their arrival sequence to the system. Also, the system can work with ambiguous or noisy data.

Later work [32] presents a methodology to undertake the motor imagery problem. A supervised version of the dFasArt (S-dFasArt) is added including the creation of different models from the learning sessions, a rule prune stage (which allows the reduction of the number of units of the models learning from the classification error on the learning sessions), and a later voting phase among the different models. This approach was successfully applied to the Data Set V of the BCI Competition.

The data processing on the BCI Competition data sets is always off-line. If the methods included on the literature were to be applied on live applications, the time constraints to produce a prediction would be a major issue to address. For instance, for the Data Set V, it is necessary to calculate the PSD function for 8 sensors and 12 frequency bands (96 features) and then apply the recognition logic 16 times per second. Moreover, there is a requirement of producing a prediction every 0.5 seconds. This computational burden requirement is not easily accommodated even on today's PCs. For on-line applications, reduction of the number of features to process is necessary.

This paper introduces a new methodology to choose the most relevant features using different approaches, being the statistic properties of the data or the relationship between the fuzzy categories which are generated on a S-dFasArt model. These methods have been applied to the Data Set V available for BCI Competition III [33] showing a reduction from 96 to 4 (96%) in the number of features required to maintain the output accuracy of the system when using a Fuzzy and GMDH (Group Method Data Handing) methodology.

The remainder of this paper is organized as follows.  Section 2 describes the data set format and structure. The methods applied are explained in Section 3.  Section 4 details the results obtained. The validation of the results and a comparison with other literature results are presented in Section 5 and finally Section 6 concludes this paper.

## 2. Data Sets Description

The work presented in this paper is based on the Data Set V available for the BCI Competition III [33] organized in 2004 by the Berlin brain-computer interface area of Berlin Institute of Technology. It is aimed to use this contest as both benchmark source and data source. For this reason, the same* rules* defined by the BCI Competition organizers have been followed, allowing us to compare the results attained by the research community with those presented on this paper. This implies using the designated sensors and maintaining the algorithms used at the preprocessing stage.

**Figure 1:**
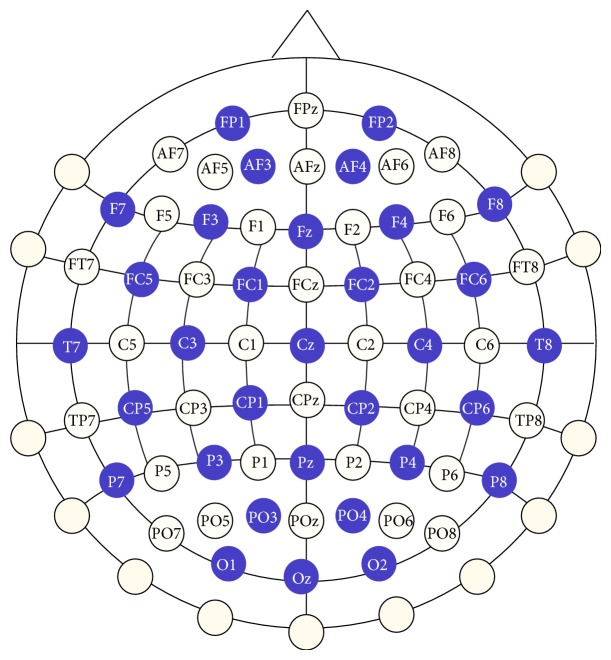
Image of the montage applying the 10-20 system convention.

The data set was provided by the IDIAP Research Institute of Switzerland and undertakes the multiclass motor imagery problem. This set was recorded by a Biosemi system using a cap with 32 integrated electrodes located at standard positions of the International 10-20 system as depicted in Figure 1. The sampling rate was 512 Hz, the signals were acquired at full DC, and no artifact rejection or correction was employed.

This data set focuses on a benchmark to classify three mental tasks [34]: left hand movement, right hand movement, and generation of words beginning with the same random letter. All sessions were obtained from healthy users with no previous EEG or mental training. The recordings were completed during the same day, each lasting 4 minutes, with 5–10 minutes breaks between them. The users were required to think about one of the three defined tasks with intervals of 15 s. Processed data from 3 of them, who recorded 4 sessions each, is used.

The precomputed sets provided only include the sensors C3, Cz, C4, CP1, CP2, P3, Pz, and P4 out of the available 32 and they are the result of several transformations of the raw data. In the first stage, the potentials recorded were spatially filtered by means of a surface Laplacian. After that, a Power Spectral Density (PSD) calculation for the frequency band between 8 and 30 Hz with a resolution of 2 Hz was performed. Being the sampling frequency 512 Hz and the records divided in windows of 1 s with an additional rate of 32 samples, an overlapping of 93.75% between windows is defined.

The computational burden of this processing can be calculated as the product of 12 different features (or different frequencies bands) per sensor by 8 channels, involving a total of 96 features per sample, yielding 49,152 features per minute.

To facilitate the understanding of the results presented in this paper, Table 1 shows the exact equivalence between the component number selected from the feature vector and the channel and frequency associated with it.

Out of the four available BCI Competition data sets per user, there are three learning data sets and a final one for testing. The learning sets are used to calculate the number of features selected by each one of the models, while the additional test session is only used at a later stage (Section 4) to validate the quality of the calculated model. Just to reiterate, the calculations presented in this paper are based on the data from the C3, Cz, C4, CP1, CP2, P3, Pz, and P4 sensors.

## 3. Methods

For the purpose of reducing the size of the features vector, a new methodology has been developed. Initially, the size of the features vector is the result of multiplying the number of channels used in the analysis by the number of frequencies considered in the PSD calculation. The classification method used is based on the S-dFasArt architecture proposed by Cano-Izquierdo et al. [32], which shows superior performance to other proposals for the multiclass motor imagery problem. It is intended that the feature selection method and the classification algorithm complement each other to maintain the overall system performance. This way, the global classification success rate can be used as a baseline, which needs to be maintained while significantly reducing the input vector.

**Figure 2:**
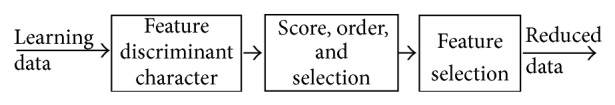
Feature selection proposed methodology.

Figure 2 presents the main stages of the selection process, which obtains a reduced set from all the initial features available in the input vector (96 in this case).

*(1) Feature Discriminant Character*. At this step, the discriminant capacity of every feature is determined. Two methods are proposed.

*(i) Statistics Method*. It is based on statistical results normally used in pattern recognition problems. This criterion only depends on the data.

*(ii) Fuzzy Criteria*. It is supported by the S-dFasArt architecture as a Fuzzy Logic System, which includes a set of rules to link fuzzy sets. Therefore, this criterion is affected by both the input data and the neurofuzzy model, which is defined by the rules calculated from the data.

*(2) Score, Order, and Selection*. For this study, a feature preselection method based on the obtained discriminant character of the data is introduced. First, the discriminant value of every feature is assessed and the feature itself is scored from 1 to 10, with 10 indicating the most and 1 the least discriminant feature. After that, all of them are sorted in descending order according to the scores given.

Then, all the scores are added according to each feature, allowing the creation of a feature classification from most to least discriminant nature. Using this ranking, a first selection of the candidate features to form the reduced vector is obtained.

*(3) Feature Selection*. In this stage, those features yielding the best performance when using the neurofuzzy classifier are selected from the candidate features set. In order to obtain the best performing subset, two different methods are proposed.

*(i) Order Selection*. By sorting the preselected features vector according to the given SCORE, (*x*_1_,…, *X*_*D*_), only *D* possible feature vectors are considered {(*x*_1_), (*x*_1_, *x*_2_), (*x*_1_, *x*_2_, *x*_3_)⋯(*x*_1_ ⋯ *x*_*D*_)}. The accuracy of every individual option is calculated by applying a *k*-fold method with the three available learning sessions and the S-dFasArt classifier. After that, the best performing features vector will be chosen.

*(ii) Group Method Data Handling (GMDH)*. This selection method evaluates the features to be added to the subset according to a Regularity Criterion (RC).

### 3.1. Feature Discriminant Character

Two methodologies, based on the training data sets, are evaluated to analyze the discriminant nature of each of the components of the feature vector: the first one is supported by applying classic statistics methods, while the second is based on the fuzzy logic interpretation of the classifier which gets created from the training data set.

#### 3.1.1. Statistics Method for Feature Selection

The framework on this research can be defined as a classification problem of *M*-dimensions in C classes. According to this premise, a set of vectors which are assumed to be “properly” classified is used and is denoted as the learning set. By using the learning set, the relative contribution for each of the features on the sampling vector to the class separability is studied. As a consequence, the properties of the statistic results from the learning vector set are calculated [35].

*γ*_*ij*_ is denoted as the variance for the *j*th feature in the *i*th class, *P*_*i*_ the a priori probability of the *i*th class, and *λ*_*j*_ the total value of the variance of the *j*th feature. The normalized variance can be defined as

(1) $$\begin{array}{c} {\tilde{\gamma }}_{ij}=P_{i}\frac{\gamma_{ij}}{\lambda_{j}}. \end{array}$$

When establishing the criteria to determine the discrimination capacity contribution of each of the features, the statistical entropy can be estimated as

(2) $$\begin{array}{c} J\left(x_{j}\right)=\overset{C}{\underset{i=1}{\sum }}{\tilde{\gamma }}_{ij}\log\left({\tilde{\gamma }}_{ij}\right). \end{array}$$

Alternative criteria to show the discriminant information of each feature can be defined as

(3) $$\begin{array}{c} J\left(x_{j}\right)=\overset{C}{\underset{i=1}{\prod }}{\tilde{\gamma }}_{ij}. \end{array}$$

This expression has a maximum value of (1/*C*)^*C*^ when all the values of ${\tilde{\gamma }}_{ij}$ are the same for a certain feature *i*. In this scenario, it can be concluded that the feature *i* does not add discriminant information and it can be dismissed.

#### 3.1.2. Fuzzy Criteria for Feature Selection

**Figure 3:**
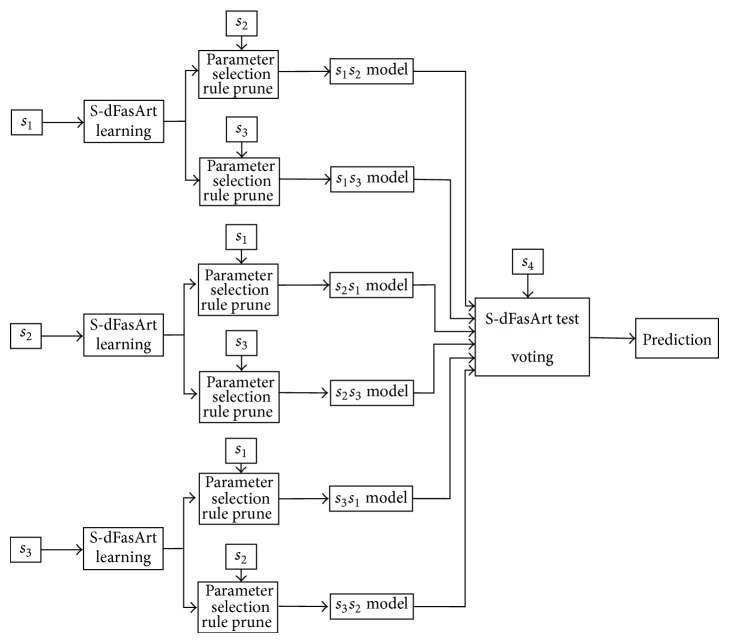
S-dFasArt classification process.

An architecture to classify EEG data applying the same benchmark as proposed in the BCI Competition Data Set V is proposed by Cano-Izquierdo et al. [32], whose output accuracy has demonstrated the ability to improve any other results published so far. The recognition system is based on the use of a neurofuzzy S-dFasArt model [14] and on a three-stage methodology, which intends to increase the utility of the three available learning sessions (Figure 3).

1. First, a learning session is used to generate a rule set defining the model.
2. After that, a different learning session is devoted to adjust the model parameters to be applied at the test stage. Then, a rule prune is performed where the rules contributing to a higher error than success rate are discarded.
3. Finally, once all the possible combinations of the three learning sessions are used for stages 1 and 2, there are six models available. For each one, 16 vectors per second are processed. Then, due to the fact that a prediction is produced every half a second only, every model contributes to 8 possible alternatives. To choose among the 48 = 6 × 8 possible predictions, a voting strategy is used where the most frequent prediction is selected.

For the purpose of feature selection, the third stage of the model is replaced by an “intermediate” model, which is defined with only three rules (each one associated with one single class). To do this, the weights defining every rule are calculated as the mean of the weights predicting the same category. The S-dFasArt model allows each class to be interpreted as a rule whose transference function is determined by the weights associated with fuzzy sets. Moreover, the rule associated with the *i* class of each feature *j* is represented by a fuzzy set *A*_*j*_^*i*^ as follows:

(4) $$\begin{array}{c} \text{IF}\;x_{1}\;\text{IS}\;A_{1}^{i}\;\text{AND}\;x_{2}\;\text{IS}\;A_{2}^{i}\;\text{AND}\;\cdots \;\text{AND}\;x_{96}\;\text{IS}\;A_{96}^{i}. \end{array}$$

Also, it is assumed that the discriminant character of each feature will be linked to the relationship between its associated fuzzy sets for two classes. If these fuzzy sets are very similar, the feature will not be very discriminant. If the fuzzy sets are clearly different, the discriminant character of the feature will increase.

For each feature, the discriminant character is obtained by comparing the corresponding fuzzy sets for two rules *i* y *k*, by using the expression:

(5) $$\begin{array}{c} F\left(x_{j}\right)=\frac{\left|A_{j}^{i}\wedge A_{j}^{k}\right|}{\left|A_{j}^{i}\vee A_{j}^{k}\right|}. \end{array}$$

A value of *F*(*x*_*j*_) near to zero denotes a very discriminant feature while a value approaching one denotes a very low discriminant feature.

### 3.2. Score, Order, and Selection

To determine the minimum number of features that can be part of the system while maintaining the output accuracy, the criteria based on the accumulated scores with regard to the total punctuation are presented. The scores are calculated by using both statistics method and fuzzy criteria. After that, the features are sorted in a descending order and the number of candidate features to be part of the model *K* is calculated as follows: *K* = min⁡{*k*} which fulfills

(6) $$\begin{array}{c} \frac{\sum_{j=1}^{k}\text{SCORE}\left(j\right)}{\sum_{j=1}^{M}\text{SCORE}\left(j\right)}>\rho . \end{array}$$

The design parameter *ρ* is adjusted to discard any feature whose SCORE value is the minimal.

### 3.3. Feature Selection

*(1) Order Selection*. The different models are being determined by selecting an increasing number of features according to the established relevance order.

**Figure 4:**
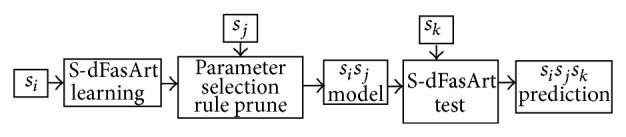
*s*_*i*_ *s*_*j*_ *s*_*k*_ model generation.

*(2) Group Method Data Handling (GMDH)*. This methodology is based on the definition of a Regularity Criterion (RC) [36], which is calculated for different candidate models, starting from single feature models. RC is considered to be the average success rate of the models for the 6 possible combinations of (*i*, *j*, *k*)  *s*_*i*_ *s*_*j*_ *s*_*k*_ as shown in Figure 4. Using single variable models as a starting point, the highest RC value is chosen. After that, a new feature is added and the model with the highest RC value is selected again. When the RC of the extended model is higher than the previous one, this one is selected as a baseline for a new iteration. When the maximum value of RC for the different models is less than the previous one, the model cannot expand and the method stops.

## 4. Results

This section summarizes the outcome of the application of the previous methodology and architecture to the BCI Competition III Data Set V database, addressing a three-class classification problem. First, the application of the statistics method is presented and the results for both Order and GMDH Selection are shown in different figures and tables. Then, the analogue information is shown for the methods based on fuzzy criteria. Section 5 joins the results of both approaches and compares them.

### 4.1. Statistics Method for Feature Selection

**Figure 5:**
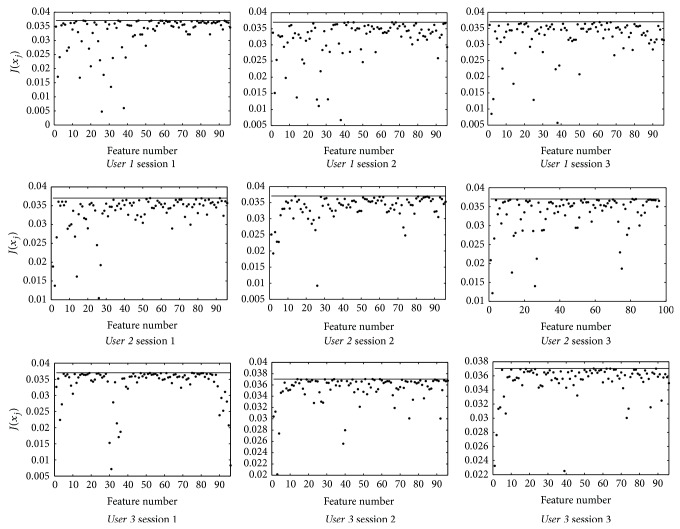
*J*(*x*_*j*_) values for the three users and sessions. The value (1/*C*)^*C*^ is represented by a solid line.

Figure 5 provides the results obtained for the three users of the BCI Competition III Data Set V database. The value of *J*(*x*_*j*_) has been calculated in a separate way for each one of the three learning sessions within the data. Given that the lower values on the figures are related to high discriminant features, the existence of a reduced number of features with a high discriminant character can be stated.

**Figure 6:**
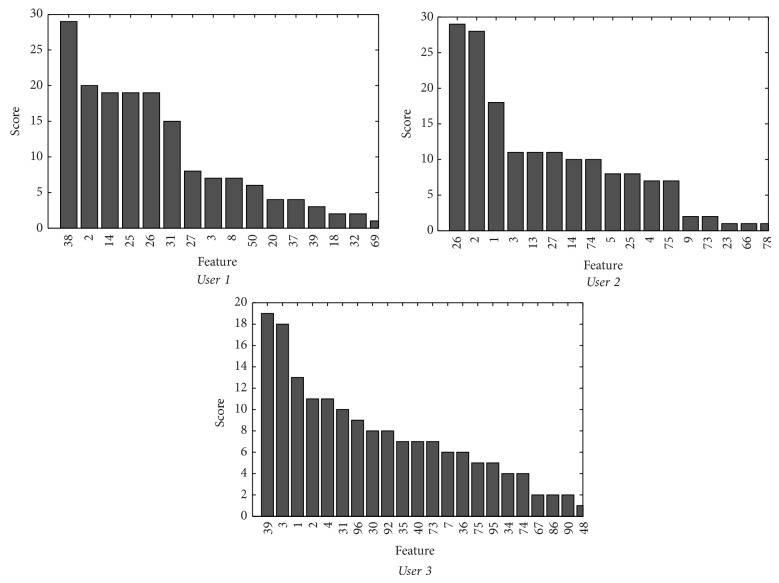
Relevance classification based on the score calculated from the discriminant nature of each feature.

To determine the most discriminant features, they have been ordered from higher to lower value of *J*(*x*_*j*_). Only the first 10 are considered and a score from 10 to 1 is assigned according to the achieved position. Once the marks from the three learning sessions are added up, the final results are gathered in Figure 6. As can be seen, the discriminant nature seems to be confined within a small number of features.  Table 2 shows the channel information and frequencies related to the ten most relevant features for each user.

The numbers of candidate features obtained after applying the 85% criteria for each of the three studied users results are *K* = 9 for* User 1*, *K* = 10 for* User 2*, and *K* = 15 for* User 3*.

#### 4.1.1. Order Selection

The results are presented in Table 3. For* User 1*, the best value of the classification success rate is achieved when using the two highest scored features. These are 38 and 2 of the input vectors, which relate to CP1-10 Hz and C3-10 Hz as shown in Table 2. Therefore, the results calculated for* User 1* can be presented as

(7) $$\begin{array}{c} X=\left(x_{38},x_{2}\right). \end{array}$$

Following the same criteria,* User 2* selected features would be as follows:

(8) $$\begin{array}{c} X=\left(x_{26},x_{2},x_{1}\right), \end{array}$$

whereas for* User 3* the features would be represented as follows:

(9) $$\begin{array}{c} X=\left(x_{39},x_{3},x_{1},x_{2},x_{4},x_{31}\right). \end{array}$$

#### 4.1.2. GMDH Selection

Table 4 shows the selected models and their RC values.

### 4.2. Fuzzy Criteria for Feature Selection

**Figure 7:**
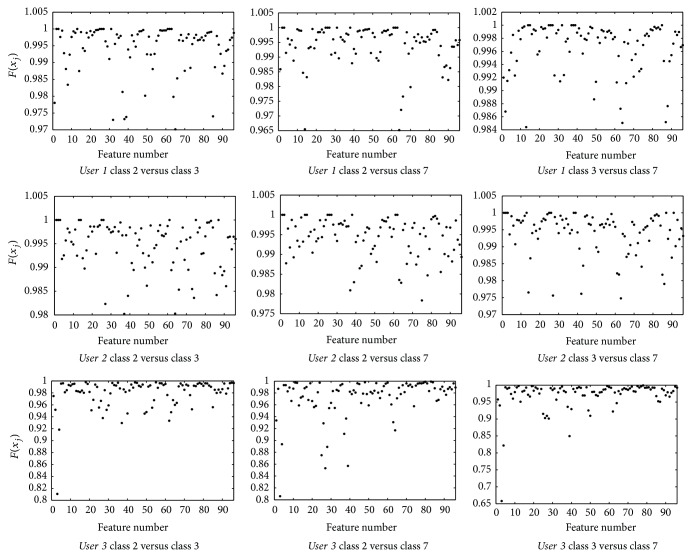
*F*(*x*_*j*_) values for the three users using session 1 for learning and session 2 for parameter adjustment and rule prune. Class “2” identifies the “LEFT” task, “3” represents “RIGHT” and “7” corresponds to “WORD”.

Figure 7 compares the discriminant character of the features for the three users by using session 1 for learning and session 2 for adjustment and rule prune. Similar results are attained when the other five combinations between the learning and the adjustment sets are calculated.

**Figure 8:**
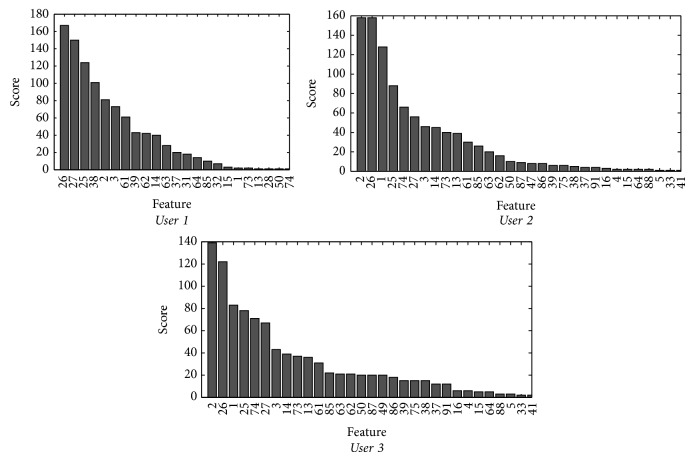
Relevance classification based on the score calculated from the discriminant nature of each feature.

If the features are sorted from the highest to lowest value of *F*(*x*_*j*_) and only the ten most important ones are selected, assigning them scores from 10 to 1 and adding them up for the six possible scenarios, the results displayed by Figure 8 are obtained.

When applying the 85% criteria on the value of *K*, *K*_1_ = 9 (*User 1*), *K*_2_ = 11 (*User 2*), and *K*_3_ = 16 (*User 3*).

The best ten channels and the frequency value attached to them for every user are provided in Table 5.

#### 4.2.1. Order Selection

Table 6 presents the different results when considering this model with an increasing number of features.

From them, the input vector for* User 1* can be presented as

(10) $$\begin{array}{c} X=\left(x_{26},x_{27},x_{25},x_{38},x_{2},x_{3},x_{61},x_{39},x_{62}\right), \end{array}$$

while for* User 2* it would be

(11) $$\begin{array}{c} X=\left(x_{26},x_{2},x_{1}\right). \end{array}$$

And for* User 3* it is as follows:

(12) $$\begin{array}{c} X=\left(x_{3},x_{74},x_{39},x_{4},x_{73}\right). \end{array}$$

#### 4.2.2. GMDH Selection

Analogously to the process followed for the statistic criteria, the GMDH method will be used with the purpose of selecting a model from a candidate feature set. In Table 7, the selection process and the final selected features are shown.

## 5. Final Validation and Discussion

It is fundamental to outline that the test set of the BCI Competition is first used in the calculations required to obtain the results presented in this section. In previous sections, only the learning session data sets are applied. In order to check the efficiency of the proposed methodology, a final stage has been performed following the method developed in [32] (Learning-Prune-Voting) with no additional parameter adjustment. The results obtained from the previous stage are shown in Table 8.

The most striking result to emerge from the data is that a reduction from a total of 96 to a range between 3 and 9 features is achieved. Interestingly, the classification success rate is maintained or even slightly improved while reducing the number of features.

Aler et al. [31] also present a feature selection process over this same data set. However, their focus is based on selecting frequency bands across all channels, so the numbers shown should be multiplied by 8 in order to be comparable with the ones above yielding 4 × 8 = 32 features for* User 1*, 2 × 8 = 16 for* User 2*, and 5 × 8 = 40 for* User 3*. As can be seen, they are much higher than the ones presented here. Another point to consider is the fact that the classification success rate presented in this paper is about 10 points higher for* Users 1 and 2*.

Similarly, another approach for feature selection is presented in [37]. In this occasion, EEG maps are created as a geometrical representation of the activity of the precomputed data of the Data Set V and only 1 frequency is selected for each user (10 Hz for* User 1*, 10 Hz for Subject 2, and 12 Hz for Subject 3). Given that data was collected by using 8 sensors, each map includes information from 8 features. Also, the amount of data used to create the map is 5 seconds, compared to the 1 second window allowed by the BCI Competition rules. Even in that advantageous situation, the classification success rate achieved is still 1.60 points lower than the Statistical and GMDH approaches.

A comparison among the classification success rate of the BCI Competition Winner, the results presented in [31, 37], and the results of this paper is shown in Table 9.

It is apparent from Table 8 that there is a subset of features appearing in all the selection methods for a certain user. For instance, features *x*_38_ and *x*_2_ are common to all models for* User 1* while *x*_26_ and *x*_2_ appear in all selection methods for* User 2* and *x*_3_ and *x*_4_ are common across the models calculated for* User 3*.

The correlation between the selected features and the users has been tested too. However, a set of common features cannot be generalized. The results show how *x*_2_ appears in all methods for* Users 1 and 2*, but it is not a part of the selected features for* User 3* by the Fuzzy selection methods. Also, it is certainly difficult to find features adopted for all users within the same selection method. As an example, for the Fuzzy + GMDH selection method, *x*_2_ and *x*_27_ are selected for* Users 1 and 2*, but they do not seem to have the same relevance for* User 3*.

Turning now to the channel position associated with the selected features (Figure 1), it can be clearly noted that important channels not only locate on the lateral area of the motor cortex, but also in the centre zone between them.

Table 10, which includes all the relevant features for all users when applying a Fuzzy + GMDH feature reduction method, clearly shows that all the selected features belong to the *α* and *β* rhythms. Also, the importance of the C3 is common to all users while C4 only appears to be useful for* Users 1 and 2*. Besides, other channels and frequencies appear to be relevant too. For instance, the *α* frequencies of the CP1 and CP2 sensors seem to be significant for* Users 1 and 3*, while *α* frequencies of P3 are important for* Users 1 and 2* as well.

This sensor selection matches neurophysiological literature as in [38], but it adds certain features which are new to this. In fact, strong evidence of the importance of the sensor positions C3 and C4 on the selection process has been found, but very little has been said about CP1, CP2, P3, or the adjacent channels. The difference on this research can be clearly motivated by the different way of constructing the data set as established in [39].

Also, the data set comprises a status which is not related to motor imagery, like it is imagining words beginning with the same random letter. This one could activate other areas of the brain and cause features not included in the previous research to appear as highly discriminant in our model.

### 5.1. Processing Time Improvement

The processing cost per feature added to the model has also been calculated for each subject.

1. At the preprocessing stage, and due to the calculations performed by the Welch periodogram PSD function, the time consumption is linear with the number of features and everyone's preprocessing cost is 1.04% of the total.
2. The neurofuzzy algorithm explained in this paper requires an increase of 9.21% of the processing time per feature during the model generation (learning and rule prune), which is very significant considering that six models are generated for each user.
3. A final 7.53% increase at the test stage for every feature added to the model is also required.

### 5.2. Unified Model for the Three Users

Table 8 shows the existence of a number of components which are selected by each individual user by all methods. For instance, being common to* User 1*, features *x*_2_ and *x*_38_ are always selected, while features *x*_2_ and *x*_26_ appear on all methods for* User 2* or *x*_3_ and *x*_4_ for* User 3*. These results have led the authors to build a unified model across all users by selecting the features *x*_2_, *x*_3_, *x*_4_, *x*_26_, and *x*_38_. The accuracy achieved by this model is shown in Table 11.

As can be found, the accuracy is slightly lower than that in the user specific models, but the reduction is only a 3.43% and the results are only improved by those shown in EEG Mapping [37], which are calculated with a 5-second window (different from the 1 s window used in the rest of the methods).

A further investigation on this field should be carried out across a larger population to determine if a reduced set of common features across the users can be found as performed by Fazli et al. [40].

## 6. Conclusion

The most obvious finding to emerge from this study is a way of drastically reducing the number of features required on the processing of the BCI systems while maintaining and even improving their classification success rate. This approach, being a three-status paradigm where only two of them are motor imagery related, has not been commonly undertaken by the literature.

The results of this investigation show that a 96% reduction of the required number of features (from 96 to 4) for a selection method based on Fuzzy and GMDH algorithms can be achieved. This translates into important time saving in computational burden when the analysis of the time consumption is performed over this simplified model.

Moreover, the methodology proposed presents a native support to multiclass problems. Most of the research papers focus on reducing channels in two tasks motor imagery paradigms. Therefore, two-class classification algorithms are an excellent tool to address the problem yielding good results in terms of the calculation time and accuracy. However, when increasing the number of classes within the problem, feature selection methods based on algorithms such as CSF, FDA, SVM, and FC require a review of the entire system and the inclusions of decision trees. In addition, the calculations need to be repeated several times in two-class space combinations, increasing the processing time and power consumption before reaching an outcome.

In contrast, the use of S-dFasArt does not require any further tuning when increasing the number of classes and the processing time remains the same due to the fact that no new calculations are being required.

It has also been shown how the user and the features selected present an important correlation. As previous studies have reported, it has been found that the *α* and *β* rhythms of the C3 and C4 channels present a big discriminant nature on the motor imagery tasks for all the studied users. Also, other *α* and *β* rhythms appear to be relevant in this scenario, which includes a nonmotor imagery task. However, the generalization capability has shown to be low, as the subset of selected features appears to be very dependent on the subject performing the task.

Further experimental investigations are needed to estimate the smallest number of common features required for the exercise presented in this paper across a larger population. An important practical implication of this would be the manufacturing of low-cost headsets with a small number of sensors. Also, the processing should be quicker as the preprocessing stage and the classification algorithm would only perform calculations on a very small set of the sampled data. Therefore, the design of devices including a reduced number of sensors could be possible. This would allow the EEG systems to be more user friendly by drastically reducing the setup time. Also, more appealing headsets compared with the current cap system could be manufactured.

In summary, it has been demonstrated that the analysis of only a few frequency bands is required. This allows an important saving in computation time and power consumption as well, which is beneficial when integrating the system, due to the fact that less processing power and memory resources are being required. The aforementioned benefits can be critical when designing applications where the available times to provide them with an output or the hardware platform are limited, for example, in applications for mobile devices.

As a consequence of the reduction in the hardware, the creation of an affordable mass market mobile system based on EEG would be possible.

## Figures and Tables

**Table 1:** Channel and frequency associated with each feature in the input vector.

Channel	Frequency (Hz)											
	8	10	12	14	16	18	20	22	24	26	28	30
C3	1	2	3	4	5	6	7	8	9	10	11	12
Cz	13	14	15	16	17	18	19	20	21	22	23	24
C4	25	26	27	28	29	30	31	32	33	34	35	36
CP1	37	38	39	40	41	42	43	44	45	46	47	48
CP2	49	50	51	52	53	54	55	56	57	58	59	60
P3	61	62	63	64	65	66	67	68	69	70	71	72
Pz	73	74	75	76	77	78	79	80	81	82	83	84
P4	85	86	87	88	89	90	91	92	93	94	95	96

**Table 2:** Features, channels, and related frequencies.

*User 1 *										
Feature	38	2	14	25	26	31	27	3	8	50
Channel	CP1	C3	Cz	C4	C4	C4	C4	C3	C3	CP2
Freq/Hz	10	10	10	8	10	20	12	12	22	10

*User 2 *										

Feature	26	2	1	3	13	27	14	74	5	25
Channel	C4	C3	C3	C3	Cz	C4	Cz	Pz	C3	C4
Freq/Hz	12	10	8	12	8	12	10	10	16	8

*User 3 *										

Feature	39	3	1	2	4	31	96	30	92	35
Channel	CP1	C3	C3	C3	C3	C4	P4	C4	P4	C4
Freq/Hz	12	12	8	10	14	20	30	18	22	28

**Table 3:** Success rate (in %) as a function of the input vector dimension (*M*) applying the statistics method and order selection. *s*_*i*_ *s*_*j*_ *s*_*k*_: *i* learning session, *j* rule prune session, and *k* test session.

*M*	*s*_1_ *s*_2_ *s*_3_	*s*_1_ *s*_3_ *s*_2_	*s*_2_ *s*_1_ *s*_3_	*s*_2_ *s*_3_ *s*_1_	*s*_3_ *s*_1_ *s*_2_	*s*_3_ *s*_2_ *s*_1_	Average
*User 1 *							
1	68.25	59.83	68.58	59.78	66.91	67.86	65.20
2	86.24	79.06	87.72	73.48	79.03	75.17	** 80.12**
3	85.96	78.40	85.26	67.66	79.41	69.15	77.64
4	84.84	79.15	87.08	66.80	81.94	65.17	77.50
5	77.05	78.40	79.74	62.44	71.51	64.71	72.31
6	74.52	77.13	79.40	54.30	73.62	66.40	70.90
7	78.17	72.18	80.13	76.63	76.90	74.00	76.34
8	77.66	76.87	80.77	63.88	77.10	78.44	75.79
9	80.10	77.53	81.25	72.65	76.84	77.69	77.68

*User 2 *							
1	44.82	62.56	57.60	46.00	59.09	49.91	53.33
2	62.56	71.44	72.90	67.34	78.91	67.63	70.13
3	68.69	75.43	72.00	68.52	75.98	63.82	** 70.74**
4	61.18	65.08	78.25	65.41	64.53	63.25	66.28
5	67.40	67.33	71.34	55.88	69.97	64.11	66.01
6	62.96	67.45	72.44	65.67	73.76	63.77	67.68
7	61.38	68.37	65.90	58.44	64.61	58.52	62.87
8	62.13	61.34	69.59	53.74	67.88	63.62	63.05
9	62.76	64.58	67.91	56.94	63.28	60.54	62.67
10	62.44	68.14	67.08	56.02	74.57	51.58	63.31

*User 3 *							
1	38.60	28.27	00.00	42.73	38.61	34.55	30.46
2	42.12	54.23	45.55	45.12	50.85	47.37	47.54
3	42.41	38.00	51.83	45.94	49.15	41.85	44.86
4	40.12	52.75	49.10	47.90	50.38	48.39	48.11
5	41.45	54.91	50.29	49.45	50.47	48.60	49.20
6	43.43	50.55	48.31	49.04	55.75	56.92	** 50.67**
7	42.06	50.32	47.30	47.37	54.47	49.53	48.51
8	40.64	48.31	44.39	45.74	50.15	49.36	46.43
9	42.35	53.27	44.88	44.16	49.85	47.78	47.05
10	40.55	58.97	43.08	44.92	53.65	50.00	48.53
11	46.05	51.29	44.27	41.91	52.95	51.66	48.02
12	47.91	54.96	44.59	50.03	48.10	54.47	50.01
13	46.69	50.73	46.48	45.71	50.64	52.25	48.75
14	44.27	55.34	50.73	48.16	44.71	52.69	49.32
15	42.59	47.75	44.30	50.82	49.47	51.52	47.74

**Table 4:** Features and RC values for the models calculated for the different users based on GMDH selection for statistics method scored data.

Features	RC
*User 1 *	
{*x*_38_}	67.96
{*x*_38_, *x*_2_}	80.12
{*x*_38_, *x*_2_, *x*_31_}	** 80.95**

*User 2 *	
{*x*_2_}	57.22
{*x*_2_, *x*_26_}	70.13
{*x*_2_, *x*_26_, *x*_5_}	** 72.72**

*User 3 *	
{*x*_4_}	49.58
{*x*_4_, *x*_3_}	51.70
{*x*_2_, *x*_3_, *x*_30_}	** 53.34**

**Table 5:** Features, channels, and related frequencies.

*User 1 *										
Feature	26	27	25	38	2	3	61	39	62	14
Channel	C4	C4	C4	CP1	C3	C3	P3	CP1	P3	Cz
Freq/Hz	10	12	8	10	10	12	8	12	10	10

*User 2 *										

Feature	2	26	1	25	74	27	3	14	73	13
Channel	C3	C4	C3	C4	Pz	C4	C3	Cz	Pz	Cz
Freq/Hz	10	10	8	8	10	12	12	10	8	8

*User 3 *										

Feature	3	74	39	4	73	27	25	1	86	26
Channel	C3	Pz	CP1	C3	Pz	C4	C4	C3	P4	C4
Freq/Hz	12	10	12	14	8	12	8	8	10	10

**Table 6:** Success rate (in %) as a function of the input vector dimension (*M*) applying the fuzzy criteria and order selection. *s*_*i*_ *s*_*j*_ *s*_*k*_: *i* learning session, *j* rule prune session, and *k* test session.

*M*	*s*_1_ *s*_2_ *s*_3_	*s*_1_ *s*_3_ *s*_2_	*s*_2_ *s*_1_ *s*_3_	*s*_2_ *s*_3_ *s*_1_	*s*_3_ *s*_1_ *s*_2_	*s*_3_ *s*_2_ *s*_1_	Average
*User 1 *							
1	49.78	55.62	39.27	40.54	54.21	58.60	49.67
2	56.98	66.04	60.17	55.39	70.28	71.65	63.42
3	65.30	60.17	65.98	59.32	67.40	59.46	62.94
4	78.08	73.73	78.78	65.14	74.02	70.53	73.38
5	78.76	73.44	80.47	78.35	77.45	69.58	76.34
6	79.71	70.19	80.63	76.86	77.85	65.97	75.20
7	79.23	78.51	78.62	58.97	79.98	68.15	73.91
8	79.07	77.79	79.48	71.62	76.50	69.47	75.66
9	80.30	79.00	80.04	66.37	81.08	81.07	** 77.98**

*User 2 *							
1	34.48	58.33	65.87	66.07	60.94	57.63	57.22
2	62.56	71.44	72.90	67.34	78.91	67.63	70.13
3	68.69	75.43	72.00	68.52	75.98	63.82	** 70.74**
4	61.43	63.31	70.56	59.50	69.53	62.33	64.44
5	61.46	70.05	67.17	59.65	63.54	53.74	62.60
6	58.06	69.70	72.87	58.41	61.83	62.07	63.83
7	68.03	65.74	65.84	58.38	45.60	61.95	60.92
8	65.01	62.24	50.60	43.98	70.95	58.67	58.58
9	68.15	66.20	62.41	48.10	66.30	58.64	61.63
10	65.67	66.41	65.15	63.59	66.26	56.45	63.92
11	52.33	67.36	68.63	58.27	56.57	55.90	59.84

*User 3 *							
1	42.50	54.61	34.65	46.41	54.47	45.59	46.37
2	41.28	36.01	40.17	46.82	49.97	46.82	43.51
3	43.87	48.60	44.22	46.90	48.01	46.06	46.28
4	44.39	53.21	41.95	48.83	51.26	48.48	48.02
5	45.17	54.38	42.88	44.89	53.91	49.45	** 48.45**
6	42.67	51.26	43.90	47.14	56.89	47.20	48.18
7	39.85	56.75	41.10	42.73	48.57	44.57	45.60
8	41.16	54.56	43.69	46.73	45.39	45.59	46.19
9	42.03	50.29	40.38	43.55	46.47	45.85	44.76
10	41.31	50.32	38.75	43.43	44.10	38.70	42.77
11	40.32	45.97	38.49	48.16	44.71	44.60	43.71
12	41.19	50.18	44.62	41.44	42.79	45.24	44.24
13	42.01	44.80	36.31	42.03	44.92	41.65	41.96
14	41.69	47.81	41.69	47.96	45.74	41.44	44.39
15	41.42	51.90	39.22	46.99	42.41	43.05	44.17
16	40.23	41.12	37.65	43.49	39.08	43.87	40.91

**Table 7:** Features and RC values for the models calculated for the different users based on GMDH selection for fuzzy criteria scored data.

Features	RC
*User 1 *	
{*x*_38_}	67.96
{*x*_38_, *x*_2_}	80.12
{*x*_38_, *x*_2_, *x*_27_}	80.77
{*x*_38_, *x*_2_, *x*_27_, *x*_62_}	** 82.22**

*User 2 *	
{*x*_2_}	57.22
{*x*_2_, *x*_26_}	70.13
{*x*_2_, *x*_26_, *x*_27_}	70.89
{*x*_2_, *x*_26_, *x*_27_, *x*_61_}	** 71.54**

*User 3 *	
{*x*_4_}	49.58
{*x*_4_, *x*_3_}	51.70
{*x*_2_, *x*_3_, *x*_49_}	** 52.41**

**Table 8:** Results for the test session.

Selection method	Model	Success rate	Number	%
*User 1 *				
None	(*x*_1_ ⋯ *x*_96_)	87.21	96	100.00
Statistic + Order	(*x*_38_, *x*_2_)	85.39	2	2.08
Statistic + GMDH	(*x*_38_, *x*_2_, *x*_31_)	87.64	3	3.13
Fuzzy + Order	(*x*_26_, *x*_27_, *x*_25_, *x*_38_, *x*_2_, *x*_3_, *x*_61_, *x*_39_, *x*_62_)	**89.95**	9	9.38
Fuzzy + GMDH	(*x*_38_, *x*_2_, *x*_27_, *x*_62_)	89.50	4	4.17

*User 2 *				
None	(*x*_1_ ⋯ *x*_96_)	82.26	96	100.00
Statistic + Order	(*x*_26_, *x*_2_, *x*_1_)	81.80	3	3.13
Statistic + GMDH	(*x*_2_, *x*_26_, *x*_5_)	81.57	3	3.13
Fuzzy + Order	(*x*_26_, *x*_2_, *x*_1_)	81.80	3	3.13
Fuzzy + GMDH	(*x*_2_, *x*_26_, *x*_27_, *x*_61_)	** 82.49**	4	4.17

*User 3 *				
None	(*x*_1_ ⋯ *x*_96_)	58.72	96	100.00
Statistic + Order	(*x*_39_, *x*_3_, *x*_1_, *x*_2_, *x*_4_, *x*_31_)	57.57	6	6.25
Statistic + GMDH	(*x*_4_, *x*_3_, *x*_30_)	** 59.40**	3	3.13
Fuzzy + Order	(*x*_3_, *x*_74_, *x*_39_, *x*_4_, *x*_73_)	52.52	5	5.21
Fuzzy + GMDH	(*x*_4_, *x*_3_, *x*_49_)	57.80	3	3.13

Average				
None		76.06	96.00	100
Statistic + Order		74.92	3.67	3.82
Statistic + GMDH		76.2	3.00	3.13
Fuzzy + Order		74.76	5.67	5.91
Fuzzy + GMDH		**76.6**	3.67	3.82

**Table 9:** Research classification success rate comparison.

Selection method	*User 1 *	Feat	*User 2 *	Feat	*User 3 *	Feat	Av.

BCI Competition Winner	79.60	96	70.31	96	56.02	96	68.65
MDLA [41]	79.68	9	66.82	17	54.59	1	67.03
SVM with evolved spatial + frequency-selection filters [31]	78.14	32	71.33	16	59.07	40	69.58
EEG Mapping [37]	85.71	8	73.80	8	64.28	8	74.60
Statistic + GMDH	**87.64**	**3**	**81.57**	**3**	**59.40**	**3**	**76.20**

**Table 10:** Selected channels and frequencies for the Fuzzy + GMDH selection method.

	Frequency (Hz)											
	8	10	12	14	16	18	20	22	24	26	28	30
*User 1 *												
C3	·	•	·	·	·	·	·	·	·	·	·	·
Cz	·	·	·	·	·	·	·	·	·	·	·	·
C4	·	·	•	·	·	·	·	·	·	·	·	·
CP1	·	•	·	·	·	·	·	·	·	·	·	·
CP2	·	·	·	·	·	·	·	·	·	·	·	·
P3	·	•	·	·	·	·	·	·	·	·	·	·
Pz	·	·	·	·	·	·	·	·	·	·	·	·
P4	·	·	·	·	·	·	·	·	·	·	·	·

*User 2 *												
C3	·	•	·	·	·	·	·	·	·	·	·	·
Cz	·	·	·	·	·	·	·	·	·	·	·	
C4	·	•	•	·	·	·	·	·	·	·	·	·
CP1	·	·	·	·	·	·	·	·	·	·	·	·
CP2	·	·	·	·	·	·	·	·	·	·	·	·
P3	•	·	·	·	·	·	·	·	·	·	·	·
Pz	·	·	·	·	·	·	·	·	·	·	·	·
P4	·	·	·	·	·	·	·	·	·	·	·	·

*User 3 *												
C3	·	·	•	•	·	·	·	·	·	·	·	·
Cz	·	·	·	·	·	·	·	·	·	·	·	·
C4	·	·	·	·	·	·	·	·	·	·	·	·
CP1	·	·	·	·	·	·	·	·	·	·	·	·
CP2	•	·	·	·	·	·	·	·	·	·	·	·
P3	·	·	·	·	·	·	·	·	·	·	·	·
Pz	·	·	·	·	·	·	·	·	·	·	·	·
P4	·	·	·	·	·	·	·	·	·	·	·	·

**Table 11:** Research classification success rate comparison.

Selection method	*User 1 *	Feat	*User 2 *	Feat	*User 3 *	Feat	Av.

BCI Competition Winner	79.60	96	70.31	96	56.02	96	68.65
MDLA [41]	79.68	9	66.82	17	54.59	1	67.03
SVM with evolved spatial + frequency-selection filters [31]	78.14	32	71.33	16	59.07	40	69.58
EEG Mapping [37]	85.71	8	73.80	8	64.28	8	74.60
Statistic + GMDH	**87.64**	**3**	**81.57**	**3**	**59.40**	**3**	**76.20**

Unified model	83.56	5	78.34	5	56.42	5	72.77

## References

[1] Ting W., Guo-zheng Y., Bang-hua Y., Hong S. (2008). EEG feature extraction based on wavelet packet decomposition for brain computer interface. *Measurement*.

[2] Lebedev M. A., Nicolelis M. A. L. (2006). Brain-machine interfaces: past, present and future. *Trends in Neurosciences*.

[3] Collinger J. L., Wodlinger B., Downey J. E. (2013). High-performance neuroprosthetic control by an individual with tetraplegia. *The Lancet*.

[4] Wolpaw J. R., Birbaumer N., McFarland D. J., Pfurtscheller G., Vaughan T. M. (2002). Brain-computer interfaces for communication and control. *Clinical Neurophysiology*.

[5] Kostov A., Polak M. (2000). Parallel man-machine training in development of EEG-based cursor control. *IEEE Transactions on Rehabilitation Engineering*.

[6] McFarland D. J., Wolpaw J. R. (2005). Sensorimotor rhythm-based brain-computer interface (BCI): feature selection by regression improves performance. *IEEE Transactions on Neural Systems and Rehabilitation Engineering*.

[7] Rosas-Cholula G., Ramírez-Cortes J. M., Alarcón-Aquino V., Martínez-Carballido J., Gómez-Gil P. On signal P-300 detection for BCI applications based on wavelet analysis and ICA preprocessing.

[8] Li Y., Pan J., Wang F., Yu Z. (2013). A hybrid BCI system combining P300 and SSVEP and its application to wheelchair control. *IEEE Transactions on Biomedical Engineering*.

[9] Panicker R. C., Puthusserypady S., Sun Y. (2011). An asynchronous P300 BCI with SSVEP-based control state detection. *IEEE Transactions on Biomedical Engineering*.

[10] Yin E., Zhou Z., Jiang J., Chen F., Liu Y., Hu D. (2013). A novel hybrid BCI speller based on the incorporation of SSVEP into the P300 paradigm. *Journal of Neural Engineering*.

[11] McFarland D. J., McCane L. M., David S. V., Wolpaw J. R. (1997). Spatial filter selection for EEG-based communication. *Electroencephalography and Clinical Neurophysiology*.

[12] Bashashati A., Fatourechi M., Ward R. K., Birch G. E. (2007). A survey of signal processing algorithms in brain-computer interfaces based on electrical brain signals. *Journal of Neural Engineering*.

[13] Mousavi E. A., Maller J. J., Fitzgerald P. B., Lithgow B. J. (2011). Wavelet Common Spatial Pattern in asynchronous offline brain computer interfaces. *Biomedical Signal Processing and Control*.

[14] Cano-Izquierdo J.-M., Almonacid M., Pinzolas M., Ibarrola J. (2009). dFasArt: dynamic neural processing in FasArt model. *Neural Networks*.

[15] Cano-Izquierdo J.-M., Almonacid M., Ibarrola J. J. (2010). Applying neuro-fuzzy model dFasArt in control systems. *Engineering Applications of Artificial Intelligence*.

[16] Lotte F., Congedo M., Lécuyer A., Lamarche F., Arnaldi B. (2007). A review of classification algorithms for EEG-based brain–computer interfaces. *Journal of Neural Engineering*.

[17] Yang J., Singh H., Hines E. L. (2012). Channel selection and classification of electroencephalogram signals: an artificial neural network and genetic algorithm-based approach. *Artificial Intelligence in Medicine*.

[18] Tam W.-K., Tong K.-Y., Meng F., Gao S. (2011). A minimal set of electrodes for motor imagery BCI to control an assistive device in chronic stroke subjects: a multi-session study. *IEEE Transactions on Neural Systems and Rehabilitation Engineering*.

[19] http://www.biosemi.com/faq/prices.htm.

[20] Schroder M., Bogdan M., Hinterberger T., Birbaumer N. Automated EEG feature selection for brain computer interfaces.

[21] Garrett D., Peterson D. A., Anderson C. W., Thaut M. H. (2003). Comparison of linear, nonlinear, and feature selection methods for EEG signal classification. *IEEE Transactions on Neural Systems and Rehabilitation Engineering*.

[22] Flotzinger D., Pregenzer M., Pfurtscheller G. Feature selection with distinction sensitive learning vector quantisation and genetic algorithms.

[23] Deriche M., Al-Ani A. A new algorithm for EEG feature selection using mutual information.

[24] Lal T. N., Schröder M., Hinterberger T. (2004). Support vector channel selection in BCI. *IEEE Transactions on Biomedical Engineering*.

[25] Wang Y., Gao S., Gao X. Common spatial pattern method for channel selection in motor imagery based brain-computer interface.

[26] Arvaneh M., Guan C., Ang K. K., Quek C. (2011). Optimizing the channel selection and classification accuracy in EEG-based BCI. *IEEE Transactions on Biomedical Engineering*.

[27] He L., Hu Y., Li Y., Li D. (2013). Channel selection by Rayleigh coefficient maximization based genetic algorithm for classifying single-trial motor imagery EEG. *Neurocomputing*.

[28] Arvaneh M., Guan C., Ang K. K., Quek C. Robust EEG channel selection across sessions in brain-computer interface involving stroke patients.

[29] Ang K. K., Chin Z. Y., Zhang H., Guan C. Filter Bank Common Spatial Pattern (FBCSP) in brain-computer interface.

[30] Ang K. K., Chin Z. Y., Zhang H., Guan C. (2012). Mutual information-based selection of optimal spatial-temporal patterns for single-trial EEG-based BCIs. *Pattern Recognition*.

[31] Aler R., Galván I. M., Valls J. (2012). Applying evolution strategies to preprocessing EEG signals for brain-computer interfaces. *Information Sciences*.

[32] Cano-Izquierdo J.-M., Ibarrola J., Almonacid M. (2012). Improving motor imagery classification with a new BCI design using neuro-fuzzy S-dFasArt. *IEEE Transactions on Neural Systems and Rehabilitation Engineering*.

[33] Berlin Brain-Computer Interface (BCI) (2004). *BCI Competition III*.

[34] Millán J. D. R. On the need for on-line learning in brain-computer interfaces.

[35] Devijver P. A., Kittler J. (1982). *Pattern Recognition: A Statistical Approach*.

[36] Sugeno M., Yasukawa T. (1993). A fuzzy-logic-based approach to qualitative modeling. *IEEE Transactions on Fuzzy Systems*.

[37] Úbeda A., Iáñez E., Azorín J. M., Sabater J. M., Fernández E. (2013). Classification method for BCIs based on the correlation of EEG maps. *Neurocomputing*.

[38] Pregenzer M., Pfurtscheller G. (1999). Frequency component selection for an EEG-based brain to computer interface. *IEEE Transactions on Rehabilitation Engineering*.

[39] Millán J. D. R., Franzé M., Mouriño J., Cincotti F., Babiloni F. (2002). Relevant EEG features for the classification of spontaneous motor-related tasks. *Biological Cybernetics*.

[40] Fazli S., Popescu F., Danóczy M., Blankertz B., Müller K.-R., Grozea C. (2009). Subject-independent mental state classification in single trials. *Neural Networks*.

[41] Mahanta M. S., Aghaei A. S., Plataniotis K. N. A Bayes optimal matrix-variate LDA for extraction of spatio-spectral features from EEG signals.

